# A Qualitative Study Assessing the Management of Predatory Journals and Their Publishing Activities: Results From the ASGLOS Study

**DOI:** 10.7759/cureus.54189

**Published:** 2024-02-14

**Authors:** Alessandro Martinino, Eloise Owen, Oshin Puri, Juan Pablo Scarano Pereira, Surobhi Chatterjee, Frank Smeenk, Sjaak Pouwels

**Affiliations:** 1 Department of Surgery, Duke University, Durham, USA; 2 Faculty of Biology, Medicine and Health, University of Manchester, Manchester, GBR; 3 Department of Microbiology, All India Institute of Medical Sciences, Delhi, IND; 4 Department of Internal Medicine, Advocate Illinois Masonic Medical Center, Chicago, USA; 5 Department of Internal Medicine, King George's Medical University, Lucknow, IND; 6 School of Health Professions Education, Maastricht University, Maastricht, NLD; 7 Department of Intensive Care Medicine, Elisabeth-TweeSteden Hospital, Tilburg, NLD

**Keywords:** time management, survey, predatory journal, electronic mail, academic spam

## Abstract

Background

Predatory journals are an emerging problem in scientific literature, as they have financial motives without guaranteeing scientific quality. Therefore, the scientific community needs to establish how this issue can be solved in the long term.

Objective

The study aims to provide information that can be used to take action against predatory journals and to guide future change.

Methods

A Google Forms (Google LLC, Mountain View, California, United States) survey was designed and disseminated between September 2021 and April 2022. Reflexive thematic analysis was used as a qualitative analysis technique in this study, with the assistance of the NVivo software (Lumivero LLC, Denver, Colorado, United States) to manage and support the analysis process.

Results

A total of 978 responses from 58 countries worldwide, achieving a response rate of 19.9%, were analyzed. Five key themes emerged regarding participants’ suggestions on techniques to cope with the detrimental impact of predatory journals: “Checking,” “Increasing education and awareness,” “Responsibility,” “Use of technology,” and “Obstacles to the solution.”

Conclusion

The outcomes of this study will help us focus and channel efforts in the future to combat predatory journals and aid us in understanding what needs to be done. We hope that this study will influence management strategies and encourage more education and awareness on a global scale.

## Introduction

Predatory journals are menacing, fraudulent phenomena that have a destructive impact on scientific research. They are a concern for many. When unscientific, non-peer-reviewed articles are published, this can potentially influence healthcare and medical decisions in a damaging way [1,2]. It is therefore important that this issue be targeted and resolved.

Predatory journals lack proper editorial and publication practices, often prioritize financial gain, and have incredibly problematic peer review practices and marketing techniques [3,4]. Diverging from the accepted worldwide standards of scientific publishing practices, using hostile, forceful communication techniques such as continual phishing emails, and having ambiguous organizational standards are all fundamental elements of predatory journals [5,6]. It is universally accepted that the presence of predatory publishing expanded with the arrival of online publishing. Jeffrey Beall first coined the term “predatory publishing” [7]. He described the devious techniques of these journals, how they deceive academics, and how they are harmful to academia. Indeed, predatory journals often fail to manage peer review properly, allowing pseudoscience to be published dressed up as authentic science.

There is no established global consensus on how predatory journals should be managed [8]. There is some advice and guidance online advising clinicians of predatory journals [9,10], but no formal consensus, plan, or body implementing change on a global scale. Predatory journals are increasingly growing in number, and consequently, a plan is needed on how we can combat them.

This qualitative study, part of the ASGLOS Study (A Global Survey on How Predatory Journals Affect Scientific Practice), aims to deliver actionable insights to combat predatory journals and inform future improvements. By examining physicians’ personal experiences and acknowledging the widespread repercussions within the academic community, the study underscores the importance of gathering varied perspectives from medical students, residents, academics, and clinicians to catalyze reform.

## Materials and methods

To accumulate the personal experiences of physicians’ mailboxes on predatory publishing, a Google Forms (Google LLC, Mountain View, California, United States) online survey was designed by two authors (AM and SP). The survey was assembled and edited by all the remaining authors. In adherence to the institutional policy of the corresponding author (SP), no IRB approval was deemed necessary for this online survey. Nevertheless, all participants explicitly grant permission for the utilization of their data for research purposes. From September 2021 to April 2022, the survey was circulated through the following: (1) the authors’ personal scientific networks; (2) The Upper Gastrointestinal Surgeons Society’s (https://www.tugsglobal.com) social media accounts on Google Groups, Facebook, Twitter, and LinkedIn; (3) The Dutch Society of Respiratory Medicine (Nederlandse Vereniging van Artsen voor Longziekten en Tuberculose); (4) The Dutch Society for Intensive Care Medicine (Nederlandse Vereniging voor Intensive Care); (5) The Dutch Society for Internal Medicine (Nederlandse Internisten Vereniging); and (6) distribution among residents and consultants in two Dutch hospitals (Saxenburgh Medical Center Hardenberg and Elisabeth-TweeSteden Hospital Tilburg; done by authors DR and SP).

The survey consisted of 34 items and was structured into four parts (Table 1). Results from parts 1, 2, and 3 were previously published [11]. This paper qualitatively analyzed the results from part 4. The question asked to participants was, “Do you have any suggestions to manage and solve the problem of predatory journals?”. Participants had the opportunity to write an unlimited number of words in an open, free text box. This study used qualitative research techniques to analyze the responses of the participants.

**Table 1:** Survey structure

Part	Section	Number of questions
Part 1	Demographics	15 questions
Part 2	Personal experience regarding predatory journals and conferences	12 questions
Part 3	Personal management regarding predatory emails	Six questions
Part 4	Suggestions to solve the problem	One question

A comprehensive analysis was conducted on 978 responses received from 58 countries worldwide, achieving a response rate of 19.9% (total possible number of participants: 4,896). A total of 160 respondents (countries with the highest response rate: India = 25%, the Netherlands = 16.2%, Greece = 8.8%, and Syria = 8.1%) out of 978 suggested one or more recommendations to solve the problem or at least to reduce the burden of academic spam (Table 2). This data will be discussed throughout this study.

**Table 2:** An overview of the qualifications of participants

Part 4	Academic qualifications					
Do you have any suggestions to manage and solve the problem of predatory journals?	Professor	Consultant	PhD student	Research fellow	Resident	Student
No	124	171	0	137	166	220
Yes	29	41	2	27	29	32

Participants who responded “Yes” were assigned a unique study identification (ID) number. Qualitative analysis was conducted by the authors using the reflexive thematic analysis technique [12,13]. This technique was used because we wanted to understand the ideas and suggestions behind the specific research question. The software NVivo (Lumivero LLC, Denver, Colorado, United States) was used to assist and manage the qualitative analysis in this study. Firstly, authors AM and EO independently read and familiarized themselves with the dataset. Following this, using the reflexive thematic analysis technique, they coded the questionnaire responses. The data was approached using an inductive approach, whereby coding and theme development were guided by the content of the responses [14]. This was done to eliminate any preconceived ideas or personal suggestions that the authors had so that the codes were only created by the dataset itself. The dataset was revisited and reread two further times to ensure saturation of coding, i.e., no further codes were created or identified. Following independent reading and coding of the dataset, the authors discussed the codes, grouping some and looking at any patterns among them. Any disagreement was resolved through discussion with a third author (SP). Throughout the reading, data immersion, and coding process, reflective memos and annotations were created. From the codes, themes were identified and evolved, and the associations behind them were interpreted and considered. Following this, these themes were refined and labeled, considering any annotations and memos that had been previously created. Themes were developed semantically, whereby themes summarized and reflected on the content of the dataset [14].

The number of participants who mentioned each theme was recorded, and a percentage was calculated from this. This data is displayed in the results section of this paper.

## Results

Five key themes emerged regarding participants’ suggestions on techniques to cope with the detrimental impact of predatory journals. These themes were “Checking,” “Increasing education and awareness,” “Responsibility,” “Use of technology,” and “Obstacles to the solution.” Explanations of the themes are presented below in the following subsections of the text; an overview of the main themes is found in Table 3.

**Table 3:** An overview of the main themes of the study

Main theme	Example quote	Percentage of participants whose responses contained themes
Checking	“To create a list of these journals, regularly update them, and make them publicly available” - Participant 20	25.6% (n = 41)
Increasing education and awareness	“Early researchers should have access to free information on predatory journals and ways to identify them. Subscription vs. open access. How to not fall into a trap. Peer mentoring can go a long way and be sustainable as well” - Participant 36	20% (n = 32)
Responsibility	“There should be an international organizing system and a regulatory body monitoring publishers” - Participant 151	15% (n = 24)
Use of technology	“Should be supported by a program for reporting such journals and emails. More so, partnering with Google or other tech companies will become useful because they can tag these websites as ‘unsafe’ or ‘unprofessional’ as the content on these sites is not actually reviewed” - Participant 98	13.7% (n = 22)
Obstacles to the solution	“It is a business model; as long as there is money to be earned on this, the problem will remain” - Participant 13	10% (n = 16)

Checking 

Checking was a predominant theme among participants, who indicated that they would benefit from being able to verify if a journal is predatory or not, with 25.6% of participants (n = 41) mentioning that having the ability to check would be a way to target the issue. Participants commented that they would like to be able to use a resource to establish how credible a journal is. More specifically, 20.6% (n = 33) of participants discussed how a list for people to utilize could be made available. A list that is public, online, user-friendly, updated frequently, and accessible were the key features participants were looking for.

*A public database of predatory journals should be made accessible so that a simple Google search should show results that indicate that the specific journal is predatory in nature* - Participant 106

*Create a list on a respected platform that would categorize these journals as predatory, which would be easy to search through* - Participant 135

Although participants generally agreed that such a list should exist, some had differing viewpoints on who should create it. Various entities were suggested, including an independent association (without any conflicts of interest), a board of academics, a legal authority, indexing agencies, or a formal research committee.

*A categorical list with all of the official, non-predatory journals from an independent association, without conflicts of interest, should be available at every email we receive for purposes of validation* - Participant 105

*Having a global registry of non-predatory journals (certified by a legal authority)* - Participant 101

Increasing education and awareness

**Figure 1:**
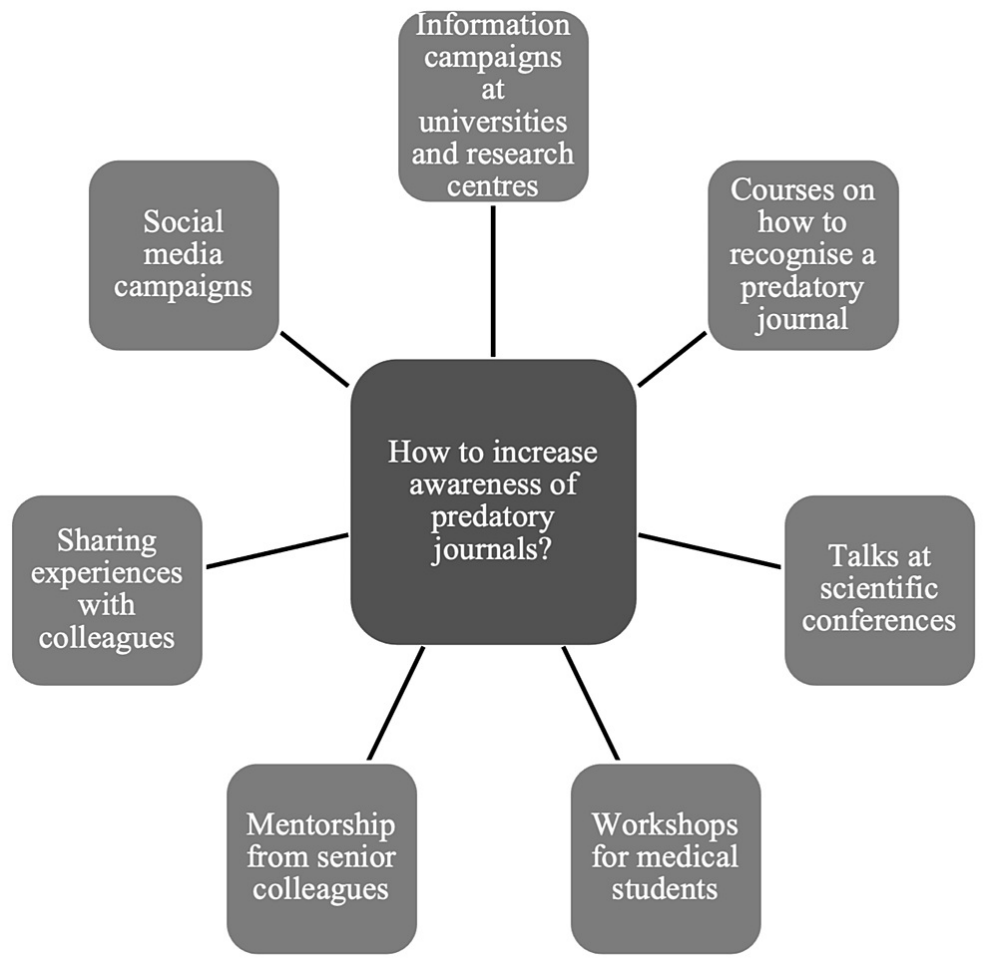
Techniques to increase awareness of predatory journals

A total of 20% of participants (n = 32) referred to increasing education and awareness of predatory journals as an important issue to address. Analysis of the answers showed that participants agreed that there is a shortfall in education regarding predatory journals and that more should be done to increase awareness of this topic. Suggestions of how this could be done are summarized in Figure 1. Participants felt strongly that increasing education about predatory journals would increase awareness, reducing their usage and attention. A mutual idea shared by many of the participants was that it is important to educate researchers and academics at the early stages of their careers. Participants discussed how, in medical school, efforts should be made to teach students about predatory journals before they get lured into the trap of predatory journals.

*Inform junior colleagues about this phenomenon repeatedly. It should be part of the curriculum in the PhD track (or even medical school)* - Participant 14

*Workshops for medical students, young doctors, and academics new to the publishing process aimed at identifying predatory journals are important* - Participant 106

It emerged that participants found it important to share their experiences of predatory journals with other researchers. In particular, sharing and speaking about emails received so others know to avoid them.

*Share the spam emails among fellow researchers so that it is easier to track and avoid them* - Participant 93

Responsibility

This theme reports on targeted ways to solve the problem of predatory journals and discusses who is culpable for making a change. This theme emerged from 15% of participants discussing who would take responsibility for this change, who would work on acting against predatory journals, and what action should be taken.

A small number of participants felt the need for predatory journals to be banned and blocked (6.9% of participants), and 5% of participants felt additionally that stronger sanctions should be implemented and that they should be punished and “criminalized.” Participants largely felt governments should be responsible for making these journals “criminal” and implementing legal sanctions.

*Put them on trial as they work against the law* - Participant 129

*High penalties by governments* - Participant 136

*The predatory journals equate to fraud, and scientific publishing should be abided by law* - Participant 36

Many of the respondents elucidated that there needs to be an organization that has the sole role of making a list of predatory journals, encouraging education and awareness of predatory journals, and policing the field in general. A total of 7.5% of participants (n = 12) suggested that there should be a governmental body policing predatory journals. There were suggestions that this organization could be a new international independent body of researchers or preexisting organizations such as the National Institutes of Health. Alternatively, some suggested that large, indexed databases should announce the names of predatory journals.

Use of technology

This theme describes how technology can be used to combat the problem of predatory journals. A total of 13.7% of participants (n = 22) articulated that the use of technology could be a solution. Participants discussed ways they would like to limit the number of spam emails from these journals. People called for a system that is automatically linked to a list of predatory journals or emails and spontaneously filters out emails from such journals, and people wished for better blocking and unsubscribing techniques.

*Having a service that auto-blocks predatory journals using their email IDs used for contact* - Participant 101

*Via cooperation between a well-known research association and email services (Outlook, Gmail, etc.) to automatically mark these emails as "predatory journals"* - Participant 118

Further suggestions of using technology to solve predatory journals would be a better way to flag these emails and report them as phishing.

*Better and easier blocking and unsubscribing methods* - Participant 69

To avoid the spamming email technique of predatory journals, it was suggested that contact details of academics and scientists on publications and conferences should not be visibly public, and hence, it would be harder for predatory journals to get in contact. 

*Limit the accessibility and visibility of email addresses of scientists in articles and conferences* - Participant 63

Obstacles to the solution

One theme that surfaced was participants discussing reasons why the problem would continue to exist. A total of 10% of participants (n = 16) spoke about a variety of obstacles and challenges to solving the problem. It was mentioned that junior trainees and students are under intense pressure to publish because this is required for further job opportunities and promotions, and therefore, they are attracted to predatory journals as an easy method of achieving publication. There was a feeling that junior researchers should be encouraged to produce high-quality work instead of a mass quantity of publications.

*Stop making it mandatory for all trainees to publish. Not all trainees need to publish* - Participant 7

*They pry on the gullible researchers, especially those who require a certain number of publications for promotion and jobs; they therefore get trapped, especially for the people who find it difficult to afford the article processing charge of the indexed journals* - Participant 49

Another obstacle to the solution, as reflected in the above quote, was that people cannot afford the article processing charge of journals, and therefore, they are tempted to use predatory journals because they are more affordable for them. Predatory journals can offer people quicker publication times and lower charges, exploiting people struggling to step into the publishing world. It was commonly suggested that if publishing in more reputable journals was more accessible and affordable, people would use predatory journals less.

*It can be very hard for some researchers to get a foothold in publishing, an avenue that predatory publishers exploit* - Participant 51

*Making indexed publications free and quick. People fall for these journals as they look to be free and quick* - Participant 37

Participants mentioned that as long as predatory journals remain profitable, they will continue to be unregulated, and as people continue to use them, they will always persist.

*It is a business model; as long as there is money to be earned on this, the problem will remain* - Participant 13

## Discussion

This is the first qualitative study establishing physicians’ opinions and ideas on predatory journals, and our findings have revealed significant results. The main goal of the ASGLOS study was to scrutinize predatory email characteristics, management, associated challenges, and potential impacts and to investigate the current issue at each academic level. This paper aimed to investigate suggestions to manage and solve the problem of predatory journals. The study answered our research question by cultivating a collection of thoughts and proposals on how this issue might be resolved. Four themes emerged as to how we can answer the problem of predatory journals: the use of technology, increasing education and awareness, a need for checking, and the need for a responsible body. One further theme was also highlighted, whereby physicians emphasized the current obstacles to the solution.

Numerous participants strongly felt that there should be a list they could check to establish if a journal is predatory. They wanted an online, accessible, and user-friendly list that they could refer to if they suspected a predatory journal. While some participants mentioned Beall’s List and suggested that a similar list ought to be created, others appeared not to be familiar with this list and thought that a list of predatory journals was a novel idea. Beall’s List, which was the first list on the internet to report predatory journals, shut down and was removed from the internet in 2017 [6]. Since then, there have been alternatives to Beall’s, including Publons’ Journal List [15], which offers users an opportunity to check whether journals are endorsed by members of the academic community. However, our study’s results indicate that most users desire a new list that is comprehensible to all and is managed by an independent association (without any conflicts of interest). It is essential that the list avoids any innate bias and is updated regularly to provide a reliable resource.

Utilizing technology to its full potential was a suggestion by many participants as a method of managing predatory journals. Participants mainly discussed how they would like to use technology to manage a large number of spam emails from predatory journals, in particular, a filtering service that connects to the list of predatory journals and automatically blocks spam emails.

Who acts and takes responsibility for predatory journals was a theme that emerged. Predatory journals are a substantial burden on the world of academia; all the solutions mentioned so far require someone to act. It was questioned by many who should take accountability for this issue and who should be the one to spearhead change. Some participants felt very strongly that predatory journals should be blocked and banned. In addition, many were emphatic that legal action should be taken against these journals. There have been cases where this has been done; in 2019, the US Federal Trade Commission won a court case against the predatory journal publisher OMICS on the basis that they used misleading claims about their conferences and publications and concealed unreasonable publication fees [16]. Analysis of the data revealed different ideas as to who should be responsible, including independent associations (without any conflicts of interest), a board of academics, a legal authority, indexing agencies, or a formal research committee. An example of a group joining together to tackle predatory journals is when Nature in 2019 facilitated a forum where 43 participants from 10 countries, representing a range of stakeholders, discussed a definition of predatory journals [17]. This indicates positive progress in combating the problem; however, our results show that more needs to be done.

It was undisputed that there needs to be more education and awareness about predatory journals. It was felt among the participants that medical students and young academics should be taught at university how to recognize a predatory journal and why predatory journals can be dangerous. This education could be done through workshops, courses, mentorship, or providing information via social media or the internet. One example of a university already doing such is The University of Edinburgh, UK, which has provided on their website “Guidelines on how to spot and avoid predatory publications” [18]. They have advised students and staff what predatory journals are, the problems and risks associated with them, and what they can do to avoid them. Our results suggest that other universities should follow suit and provide information on predatory journals to their students and staff to increase awareness.

In addition to increasing education about predatory journals among those early in their careers, it was also suggested that more can be done to increase awareness among established researchers. The “Think. Check. Submit” campaign is an organization that is already paving the way to combat predatory journals [19]. It helps researchers recognize trusted journals and publishers for their research by providing a range of tools and resources. However, none of our participants mentioned this campaign, so potentially, more needs to be done to increase awareness of it. Similarly, the Committee on Publication Ethics (COPE) has released information to increase awareness of predatory journals [20] and has collaborated with the “Think. Check. Submit” campaign. By publicizing this information, the committee and campaign would assist in increasing awareness.

Despite our research question asking for suggestions to solve the issue, we identified reasons why physicians feel the problem will remain and why this could hinder solving predatory journals. Aspects included that predatory journals will always remain because they are business and money-making schemes and will continually adapt to lure researchers in. In addition, another obstacle to the solution was the pressure to publish in the early stages of medical careers to gain further job opportunities. Physicians in our study commented that early-stage researchers may be knowingly publishing in predatory journals as they think this will advance their job opportunities. An example of this is in the UK, wherein in 2022, candidates applying for core surgical training (typically two years after graduating from university) will gain an extra six points in their application if they are the first author of one or more PubMed-cited publications [21]. Participants felt that this tremendous pressure to publish was one reason why junior researchers and clinicians might publish in predatory journals - to increase their likelihood of promotion. Therefore, only by changing the attitudes of employers and the community to reward high-quality work over quantity will we target this obstacle to the solution.

Strengths and limitations of the study

This study qualitatively analyzed the answers to part 4 of the ASGLOS survey. The benefit of conducting qualitative analysis was that it helped us explore the participants’ needs, understand the reality of predatory journals, and comprehend what interventions could be done to resolve the problem. By giving participants the opportunity to explain their opinions and suggestions on how to manage predatory journals, we were able to discover new ideas and truly recognize the negative impact that predatory journals have on the academic world.

This study accumulated the personal experiences of medical students, residents, research fellows, PhD students, consultants, and professors active in scientific research. An advantage of this study was that it had a large sample size (n = 160). Furthermore, because it was an international study, participants were from a range of countries and at different stages in their careers. This adds strength to the study because it allows us to obtain a rich dataset representative of all opinions. One limitation of this analysis was that we only analyzed one question, which was part of a more extensive study. More open-ended, free-text questions could have been asked to obtain a richer dataset to analyze. In addition, a future study could benefit from interviews with participants and analyzing the transcripts because this could provide deeper, more in-depth data. Alternatively, a Delphi study could be used in the future to establish a consensus opinion [22]. Additionally, our paper may also be subject to a bias in the response rate. This potential bias could affect the validity of our findings by skewing the data toward those who chose to participate, potentially omitting diverse perspectives from those who did not respond.

Predatory journals do not just affect the medicine or biomedical fields. Technology, engineering, and legal research fields are also heavily affected [23]. The InterAcademy Partnership (IAP) is a worldwide group of science, engineering, and medical academies working together. In March 2022, they published a report alerting the research community to the dangers of predatory journals, exploring their prevalence and impact [24]. One limitation of our research is that it just focused on the medical field; however, since this is an interdisciplinary issue, future research may benefit from consulting different fields in the academic community.

## Conclusions

There is no doubt about predatory journals’ damaging impact on the scientific world. It is a crucial issue we must fight in the medical field because the effects are harmful. It can lead to the publication of false, inaccurate information, which may ultimately affect patient care, health, and outcomes. By listening to physicians’ opinions regarding predatory journals, we learned about various obstacles to finding a solution and the reasons why the problem remains. Understanding the core of the problem will help align future changes. The findings from this study have also revealed suggestions and ideas to solve the problem of predatory journals. This study has found that, specifically, the need for checking, the use of technology, increasing awareness and education, and forming a responsible body are important answers to solving predatory journals. The outcomes of this qualitative study will help us focus and channel efforts in the future to combat predatory journals and aid us in understanding what needs to be done. We hope that the results from this study will influence management strategies for predatory journals and encourage more education and awareness on a global scale.
